# High Redundancy as well as Complementary Prey Choice Characterize Generalist Predator Food Webs in Agroecosystems

**DOI:** 10.1038/s41598-018-26191-0

**Published:** 2018-05-23

**Authors:** Eve Roubinet, Tomas Jonsson, Gerard Malsher, Karin Staudacher, Michael Traugott, Barbara Ekbom, Mattias Jonsson

**Affiliations:** 1 Department of Ecology, Swedish University of Agricultural Sciences, Uppsala, Sweden; 2 Ecological Modelling group, School of Biosciences, Skövde University, Skövde, Sweden; 3 Mountain Agriculture Research Unit, Institute of Ecology, University of Innsbruck, Innsbruck, Austria

## Abstract

Food web structure influences ecosystem functioning and the strength and stability of associated ecosystem services. With their broad diet, generalist predators represent key nodes in the structure of many food webs and they contribute substantially to ecosystem services such as biological pest control. However, until recently it has been difficult to empirically assess food web structure with generalist predators. We utilized DNA-based molecular gut-content analyses to assess the prey use of a set of generalist invertebrate predator species common in temperate agricultural fields. We investigated the degree of specialization of predator-prey food webs at two key stages of the cropping season and analysed the link temperature of different trophic links, to identify non-random predation. We found a low level of specialization in our food webs, and identified warm and cool links which may result from active prey choice or avoidance. We also found a within-season variation in interaction strength between predators and aphid pests which differed among predator species. Our results show a high time-specific functional redundancy of the predator community, but also suggest temporally complementary prey choice due to within-season succession of some predator species.

## Introduction

Ecosystem functioning is, to a large extent, determined by food web structure^1^. A good understanding of the structure of food webs and their temporal variation is therefore important to disentangle the drivers of various ecosystem functions and to be able to predict their response to perturbations^2–4^. So far, however, comprehensive descriptions of food webs with spatial and/or temporal resolution are few (see e.g.^5–8^). Furthermore, studies examining temporal variation in food web structure of systems that include cryptic feeding interactions, or predators with difficult-to-identify prey remains, are rare (but see^9^). In arthropod host-parasitoid food webs, these constraints are usually dealt with by means of host collection and parasitoid rearing^7,10^, although these approaches still have some important limitations (e.g., they do not allow resolution of links between primary and hyperparasitoids)^11^.

Molecular gut-content analyses (MGCA) are radically improving our understanding of the trophic structure of food webs by identifying prey DNA in predators’ guts^11–14^. The use of these techniques has increased the number of trophic links detected in comparison to traditional techniques, and thus allows a better description of the structural properties and species roles in arthropod food webs^15^. So far, however, these analyses have mostly included limited subsets of predators and prey (e.g.^16–18^, but see^19^). In addition, there is a lack of studies on the temporal variability in diet composition of interacting predator species (but see^9^), or that attempt to determine if predators make active prey choices in complex prey communities. These limitations result in important knowledge gaps in our understanding of how larger sets of species interact and affect the overall function of predation over time in arthropod communities.

Compared to many other systems, agricultural systems are simple and generally well-known in terms of species composition and trophic complexity, at least aboveground. They offer an opportunity of studying predation by most of the predator taxa present. In addition, understanding which predator eats what prey, during which period of the season in agricultural systems is vital for understanding the ecosystem service of biological pest control. Species abundances in agroecosystems are influenced by significant within-seasonal changes and disturbances due to field management, which has potential implications for both prey availability and predators’ preferences for different prey. This might in turn affect the food web structure, including its degree of specialization^20^, and the role of generalist predators as regulators of pest populations. More specifically, this could affect the randomness of predator diets and the resource use strategy of the predator community, such as its functional redundancy and its temporal and/or spatial resource complementarity^21^, and therefore the overall level of predation over time. In order to ascertain the extent of predator specialization and the level of active prey choice, a comparison of the frequencies of feeding interactions (i.e. number of predation events between a predator species and a prey species) to prey availability is necessary. So far, this has been based exclusively on comparing observed diet composition of predators to field estimates of prey densities (e.g.^22^), although prey density may not be an accurate reflection of their availability to predators. Furthermore, field abundance estimates of diverse prey communities usually rely on several different sampling methods, each with different biases (e.g.^23^), which makes density estimates for different prey groups difficult to compare. Here we attempt to circumvent these pitfalls by using a null model approach where we compare the observed distribution of predator-prey feeding interactions to that expected if feeding interactions were randomly distributed among predators and their potential prey. This allows us to measure the ‘temperature’ of individual trophic links, i.e., the deviation of observed frequency of a predator-prey link from null model expectation to identify specific predator-prey links that cause non-random structure. Such feeding interactions are potentially driven by prey preferences (or active dismissal) of the predator and/or assortative (or disassortative) microhabitat preferences of predator and prey that affect prey availability positively (or negatively)^24^.

In this study, we investigate prey preferences of eight common generalist invertebrate predator species using DNA-based MGCA targeting fifteen inter- and intraguild prey. Ten spring barley fields were surveyed at two successive time periods during the cropping season (tillering ‘Early period’ and heading stages ‘Late period’). These periods correspond to when generalist predators can impact the field population of the bird-cherry oat aphid, *Rhopalosiphum padi* (Linnaeus), one of the key pests of cereal crops in Sweden^25,26^ and worldwide^27^. The Early period typically coincides with the aphid colonization phase and has low overall herbivore abundance and low structural complexity of the crop, and the Late period is usually characterized by peak aphid populations, high overall herbivore abundance and high structural complexity of the crop^25^. At both time points we (i) estimate the level of specialization of predator-prey invertebrate food webs at the network as well as species level, and (ii) analyse the potential (non-) randomness of predator diets in relation to a null model. We hypothesize to find a high functional redundancy of the generalist predator community, defined as a low level of food web specialization.

Given the biotic and abiotic changes occurring during the cropping season in agricultural systems, we expect a decrease in food web specialization as the season progresses which should reflect changes in the distribution of feeding interactions at the predator species-level, possibly driven by an overall increase in prey abundances (H1). The distribution of feeding interactions is furthermore expected to be more random early in the cropping season as a result of predators being able to make fewer active choices when prey is scarce (H2), which should additionally be supported by individual link temperatures significantly differing more from zero late compared to early in the cropping season.

## Results

### Prey detection frequencies in generalist predators

**Figure 1:**
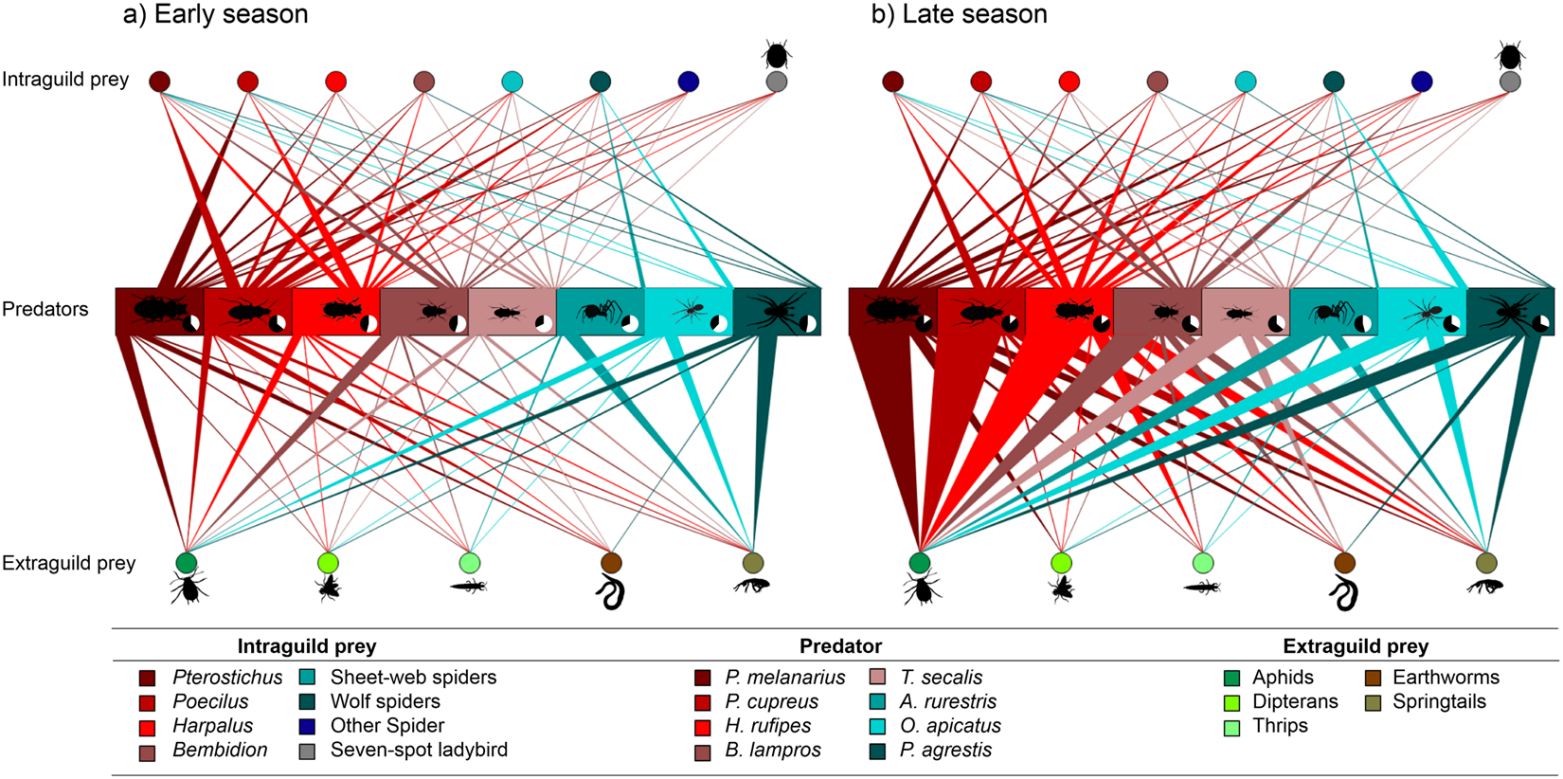
Predator-prey food web in the (**a**) Early and (**b**) Late period of the cropping season. We used the total (across 10 fields) proportion of generalist predators (middle row) screened that were positive for extraguild prey (aphid, dipterans, thrips, earthworms, springtails; bottom row) and intraguild prey (*Pterostichus*, *Poecilus*, *Harpalus*, *Bembidion*, sheet-web spiders, wolf spiders, other spiders, seven-spot ladybird; upper row) to generate these webs. The small circle by each animal shows the proportion screening negative for all prey types (white). A total of 3,680 generalist predators belonging to eight species were screened (carabids: *Pterostichus melanarius, Poecilus cupreus, Harpalus rufipes, Bembidion lampros, Trechus secalis* and spiders: *Agyneta rurestris* [Linyphiidae], *Oedothorax apicatus* [Linyphiidae] and *Pardosa agrestis* [Lycosidae]). Per field average and variability is shown in Fig. S1. Figure initially prepared with ‘Food Web Designer’^49^. Prof Klaus Birkhofer is acknowledged for drawing the invertebrates.

Out of the 15 prey taxa targeted by the MGCA, all but the intraguild prey lacewings and *Pachygnatha* (family: Tetragnathidae) were detected in the guts of the eight generalist predator species analysed. Across both sampling periods, 36% of the predator individuals screened were negative for all prey taxa, 35% were positive for one prey taxon, and the remaining 29% were positive for more than one prey taxon. Although a large majority of the detected feeding interactions were present in both sampling periods (Fig. 1) the proportion of generalist predator individuals that were negative for all prey targeted by the MGCA (i.e., with ‘empty gut’) decreased from Early (54.6% ± 4.3, average ± SEM) to Late period (27.8% ± 4.3) (ANOVA, df = 14, P < 0.001). This was mainly driven by an increase in detection frequencies, of most extraguild prey taxa in most predator species (Figs 1; S1a), while a similar trend could not be seen for intraguild prey (Figs 1; S1b). In particular, the detection frequencies of aphids increased strongly from the Early to the Late period in all generalist predator species (Figs 1; S1a). A similar pattern was observed for springtails, detected in carabid predators. Intraguild predation was more prevalent in carabids than spiders, and was unidirectional across these two groups, i.e., carabids fed on spiders but spiders rarely fed on carabids (Figs 1; S1b).

### Food web specialization and variations within the cropping season

**Figure 2:**
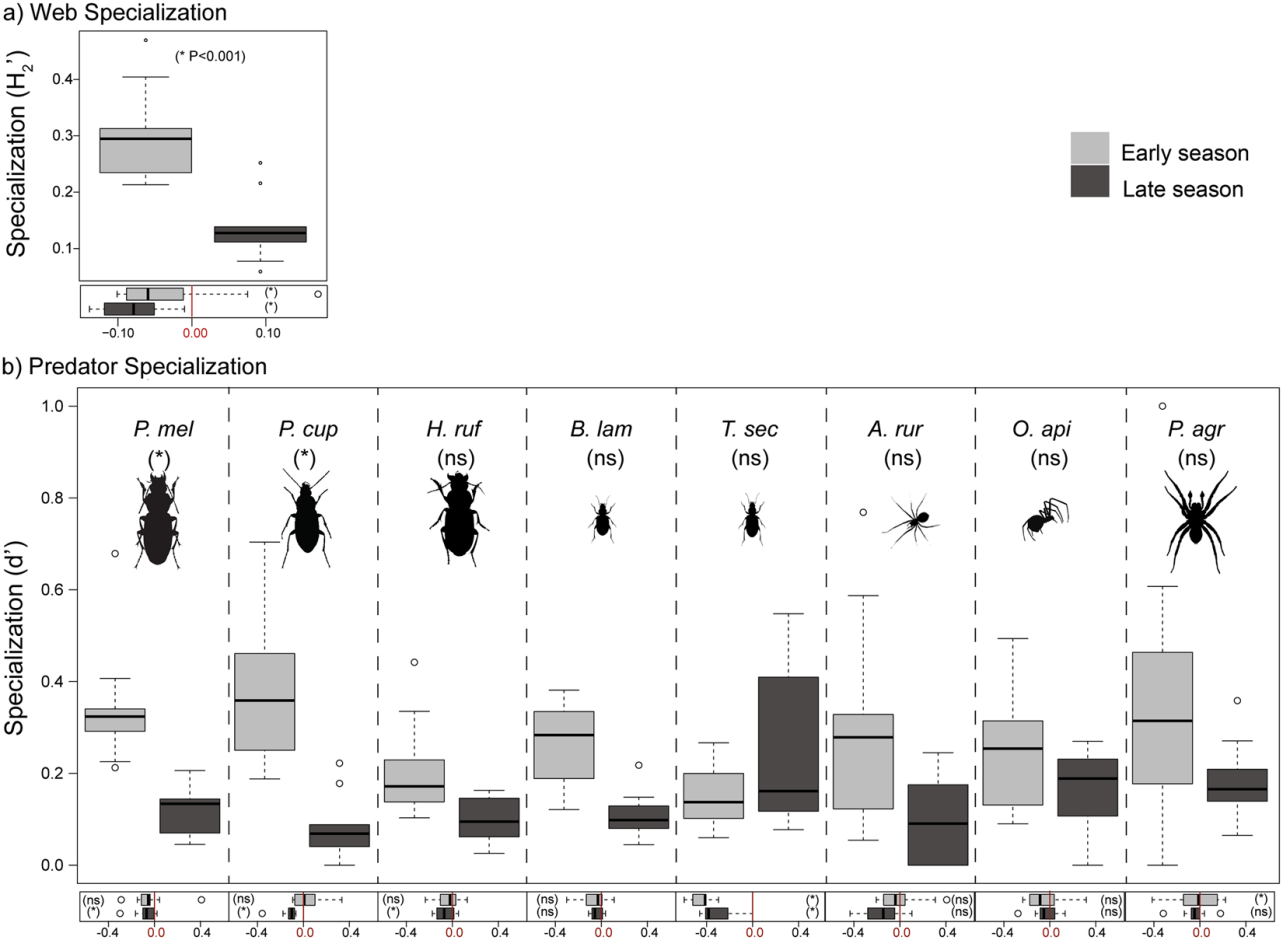
Network- (**a**) and species- (**b**) level specialization (upper boxplot) and deviations in observed metrics from null model expectations (lower boxplot, see methods) in the Early and Late period (light and dark grey boxes respectively. Boxplots show medians (horizontal line), 25th and 75th percentiles (upper and lower box limits), extreme observations (bars) and data identified as outliers (dots). *And *ns* denote whether there was a significant difference in the (i) variation within the cropping season, at α = 0.05, in the observed food web metric (reported in parenthesis inside the upper boxplot), and (ii) deviation from null model expectation of the observed food web metric (reported in parenthesis inside the lower boxplot). There was no significant variation within the cropping season observed in the deviation from null model expectations for specialization at neither network- nor species level. Prof Klaus Birkhofer is acknowledged for drawing the invertebrates.

In general, the predator-prey food webs had low levels of specialization (${H^{\prime} }_{2}$ = 0.22 ± 0.02, across-season mean ± SEM), with a significant decrease from the Early to the Late period (Fig. 2a, ANOVA, P < 0.001; Table S4). Similarly, specialization of predator species was affected by period within the cropping season (Fig. 2b) and decreased significantly from Early to Late period across all species, with variation in the within-season trend depending on predator identity (ANOVA, Periods x Species, P < 0.001, Table S4). Variation in the degree of specialization at the species level within the cropping season was higher for carabids (except for *T. secalis*) than for spiders.

### Randomness of feeding interactions

At the network-level, the deviation of web specialization from null model expectations varied with site and within the cropping season: web specialization differed from random at three sites in the Early period and seven sites in the Late period (Table S5). However, across all sites, web specialization deviated from null model expectations (Binomial test, P < 0.05 in both the Early and Late period, Table S5). Predator-prey food webs tended to be less specialized than the null model expectation (Fig. 2a, lower boxplot).

At the species level, the deviation from null model expectations of predator specialization (*d’*) also varied with site, predator species, and within the cropping season (Fig. 2b, lower boxplots), with between zero and five or six sites showing specialization that differed from random in the Early and Late periods (Table S7a). Across all sites, contrary to web specialization, predator specialization generally did not deviate from random in the Early period (Binomial test, P > 0.05 for all predator species except *T. secalis* and *P. agrestis*: P < 0.001, Table S7). By contrast, in the Late period, specialization of all carabids except *B. lampros* was significantly lower than expected (Binomial test, P < 0.05, Table S7), while for spiders it did not deviate from random. The extent to which the observed predator specialization deviated from randomness was not significantly affected by period (ANOVA, P = 0.068, Table S6), but it did vary according to predator species (ANOVA, P < 0.001).

### Temperature of links

**Figure 3:**
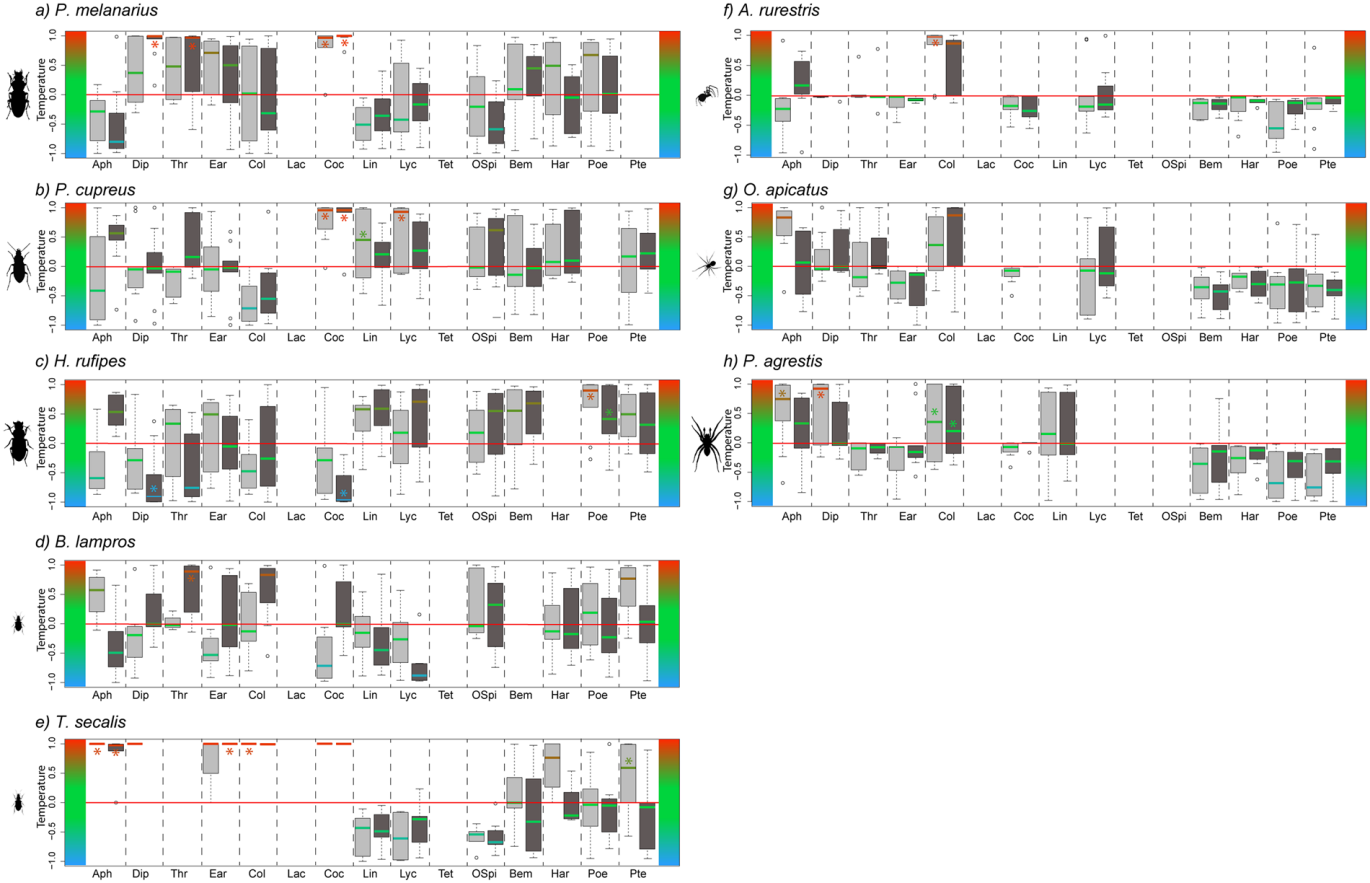
Temperature of predator-prey links in the Early and Late period (light and dark grey boxes respectively) for eight generalist predators (carabid beetles: (**a**) *Pterostichus melanarius*, (**b**) *Poecilus cupreus*, (**c**) *Harpalus rufipes*, (**d**) *Bembidion lampros*, (**e**) *Trechus secalis* and spiders: (**f**) *Agyneta rurestris* [Linyphiidae], (**g**) *Oedothorax apicatus* [Linyphiidae] and (**g**) *Pardosa agrestis* [Lycosidae]) and the 15 prey targeted by our molecular assays (extraguild prey: aphids, dipterans, thrips, earthworms, and springtails; and intraguild prey: lacewings, seven-spot ladybird [Coc], sheet-web spiders [Lin], wolf spiders [Lyc], *Pachygnatha* [Tet], other spiders, *Bembidion*, *Harpalus*, *Poecilus* and *Pterostichus*). Boxplots show medians (horizontal line), 25^th^ and 75^th^ percentiles (upper and lower box limits), extreme values (bars) and data identified as outliers (dots) of link temperature in 10 fields. Colours show the ‘temperature’ of the links, i.e., the frequency that an observed link is greater or smaller than expected (see methods). Red, green, and blue denote warm (more observed than expected by 1000 null models), neutral (as expected), and cool (less observed than expected by 1000 null models) links, respectively. *Denotes links statistically warmer (>0.95) or colder (<0.95) than expected by the null models across fields (see methods). Prof Klaus Birkhofer is acknowledged for drawing the invertebrates.

There was a lot of variability in link temperatures among fields, so that only a few link temperatures were significantly warm or cold (Binomial tests, P < 0.05, Fig. 3). In general, the temperature of predator-prey links (i.e., the deviation of the frequency of observed feeding interactions from neutrality if feeding interactions had occurred randomly) varied according to predator species and prey taxa (ANOVA, predator species × prey taxa: P < 0.001, Table S8) but did not vary according to period within the cropping season (ANOVA, P = 0.586). However, the temperature of particular trophic links, such as links to aphids, tended to vary within the cropping season for most predators. Links to intraguild prey were generally warmer for carabids (Fig. 3a–e) than for spiders (Fig. 3f–h). Furthermore, prey choice and link temperatures of the three spider species varied less than among the carabid species, and more links appeared to be neutral (i.e., did not deviate much from null model expectations) than for carabids. More specifically, all spiders had warm links to springtails, but cool links to earthworms, the seven-spot ladybird and to carabid intraguild prey. Warm links to aphids were detected for *O. apicatus* (Early period) and *P. agrestis* while for *A. rurestris* they changed from cool in the Early period to warm in the Late period.

For carabid predators, the temperature of links to extraguild prey varied according to predator species. In both periods, *P. melanarius* had warm links to thrips and earthworms, and cool links to aphids. Links to dipterans were warm for *P. melanarius* and cool for *H. rufipes. Poecilus cupreus* and *H. rufipes* had cool links to springtails. *Trechus secalis* had warm links to most extraguild prey. *Bembidion lampros* had cool links to earthworms in the Early period, and warm links to thrips and springtails in the Late period. Links to aphids were warm in the Late period for *P. cupreus*. Links to aphids changed from cool in the Early period to warm in the Late period for both *H. rufipes* and *B. lampros*. In general, the temperature of links for carabids to intraguild prey did not vary within the cropping season: *P. melanarius* had warm links to *B. lampros* and to the seven-spot ladybird while links to sheet-web spiders and other spiders were cool. *Poecilus cupreus* had warm links to many intraguild prey taxa. *Harpalus rufipes* had cool links to the seven-spot ladybird but warm links to sheet-web spiders, *Bembidion*, and *Poecilus*. By contrast, *B. lampros* and *T. secalis* had cool links to sheet-web spiders and wolf spiders. In addition, in the Early period only, links were warm from *T. secalis* to *Harpalus* and *Poecilus*, and from *B. lampros* to *Pterostichus;* whereas links from *B. lampros* to the seven-spot ladybird were cool.

## Discussion

In this study, we examine the variation of generalist predator-prey food webs within the cropping season in replicated spring barley fields. The level of specialization of the predator-prey food webs was generally low at both network- and species-levels and significantly decreased during the cropping season, which supports our first hypothesis (H1). Food webs differed from random expectations throughout the cropping season, but there was no systematic pattern of within-season change in the magnitude of deviation from randomness (not supporting H2). Similarly, there was no within-season trend in the deviation from random expectations for the predator-prey interaction strengths (thus not supporting H2). Variation in link temperature depended on predator species and prey taxa, with warm links being most common between carabid predators and intraguild prey. Below, we discuss these results in the context of our two hypotheses and highlight the potential implications for the functional role of arthropod predators in agroecosystems.

There were significant differences in the specialization of our predator-prey food webs between early and late periods in the cropping season. Specifically, the degree of specialization at network- and species-level decreased during the cropping season, from low to extremely low levels of specialization. The degree of specialization at the network level later in the cropping season was of the same magnitude as those documented in ant-plant mutualistic networks^28^ and in second to first order parasitoid networks, but much lower than those recently observed in other antagonistic networks (e.g., plant-herbivores^29^; host-first order parasitoids^30^). The taxonomic resolution of prey (or plant resources) in these studies differ, however, which makes comparisons of the degrees of specialization difficult. Despite this caveat, the observed low level of specialization suggests a high level of niche overlap and therefore a high functional redundancy among generalist predators in agricultural fields^31–33^.

The within-season variation in network specialization is likely explained by prey availability dynamics (i.e., changes in abundance and/or life stage of prey species), as herbivore abundances in general increased from early to late in the cropping season (Fig. S2). High functional redundancy suggests that a high diversity of predators may not be needed to provide effective pest control in the short term (as opposed to higher specialization and complementarity^34^). In the long term, it can have a positive effect on the stability of the function by acting as an insurance against perturbations or variations in predators abundances^21,35^.

In addition to a low level of specialization at both network- and species level, the structure of the predator-prey food webs at the network level differed from random expectations. High predator redundancy in the network structure thus appears, at least partly, to be driven by non-random processes. Preferences in prey and phenological or spatial mismatches^36^ are examples of mechanisms that can explain the non-random structures that are often observed in agricultural systems (e.g.^37^). However, contrary to our hypothesis, there was no within-season trend in the direction or magnitude of deviation from random. At the predator species levels, however, specialization did not differ from random early in the cropping season, which may be explained by a lower sample size than at the network-level.

For some links (e.g., Early period: *P. cupreus* and *A. rurestris* ->springtails and Late period: *H. rufipes* ->aphids or *P. melanarius* ->thrips) there was qualitative as well as quantitative consistency in link temperatures across fields. Most links, however, showed large inter-field variability in the value, and sometimes also the sign, of link temperatures. This inter-field variability is likely not due to sampling effects, as the variability across fields did not change when running the same analysis only including the predator species for which the number of collected individuals per field and period was above a minimum threshold (n > 10, 15 or 20). Such variability might instead be due to differences in prey and predator abundances among fields, which could modify the strength of predator-prey links^38,39^. Nevertheless, there was no obvious within-season trend in link temperature, i.e., in the deviation of the strength of observed links from neutrality. This does not support our second hypothesis, which predicted more active prey choices (which here would be seen as a greater number of both warm and cold links and/or more extreme temperatures of already warm and cold links) with increasing prey availability later in the cropping season (H2). However, we found more warm links for carabids than for spiders, primarily due to a high proportion of warm links between carabids and intraguild prey. In addition, we found warm links to extraguild prey that reflect predator feeding preferences already suggested in previous studies. For example, we found that spiders had warm links to springtails (see e.g.^22^), and large carabids had warm links to earthworms^40^. Springtails have been shown to be a high quality food source for spiders^41^, which together with our result emphasizes the importance of these decomposers for sustaining generalist predator populations.

Compared with other prey, link temperatures between predators and aphids were particularly dependent on predator species and on period within the cropping season. We show that links between aphids and spiders, and between aphids and small carabid species (*B. lampros* and *T. secalis*) are warm during times when cereal aphids colonize fields, while links between aphids and large carabids *(P. cupreus* and *H. rufipes*) are warm once aphid populations were established. This result suggests a within-season succession of predator species in terms of feeding activity on aphid prey that could have implications for biological control. In addition, we found that the large carabids (*P. cupreus* and *H. rufipes*) actively prey on spiders, whereas intraguild predation by spiders on carabid prey was rare. These results most likely reflect spiders avoiding carabid prey due to difficulties in handling, and potentially a high vulnerability to attack from carabids^9,17^. Thus, if large carabids happen to dominate arthropod communities early in the cropping season and actively prefer non-aphid extraguild- and intraguild prey, this might hamper the service of biological control. On the other hand, if large carabids only dominate in late cropping season this might strengthen the service of biological control by providing a temporal complementarity in prey use of important herbivore predators. Further experiments analysing the effect on biological control would be needed to confirm this scenario, in which case pest management strategies could optimize biological control by taking the phenologies of different species into account.

We acknowledge, however, that our null model assumption that all prey items in a given area were equally available to all analysed predator species in that area may not be entirely realistic. Differences in hunting strategies between generalist predator species, microhabitat preferences of both predator species and prey taxa, and matching predator-prey traits such as body size might lead to varying prey availabilities. Additionally, we acknowledge that the temperature of links (and the level of specialization) may partly depend on the relatively coarse taxonomic resolution (genus, family, or higher level) used in our molecular assays. Prey identified to order level (e.g., dipterans) is a more heterogeneous group of species than prey identified to family level (e.g., aphids, sheet-web spiders), so preferential predation towards a subgroup is more likely to have occurred at family level than at order level. The temperature of links to prey identified at higher levels is thus likely to be variable, and predator choice might be difficult to observe. Once more highly resolved assays, or barcoding, can be deployed, future studies can address this caveat and challenge the results presented here. Additionally, the use of our null model approach together with estimates of field densities could be the next step to provide more insights into predator choice and what drives predator-prey feeding interactions.

Our study describes the structure and within-season variation of generalist predator-prey invertebrate food webs in an agricultural system. We used a combination of molecular analyses, null models and link temperatures to analyse the food webs. Such a combination of methods improves our understanding of trophic feeding interactions and of the (non)-random processes involved in shaping the food web structure, and can be applied to various other ecosystems. We found a low level of specialization at both the network- and predator species level, especially during the Late period, as well as significant within-season variability in link temperatures among predators. We furthermore found our webs to be less specialized than null-model expectations and many link temperatures to differ from zero, emphasizing the non-random, but variable, nature of trophic feeding interactions. Together, these results have implications for ecosystem services in agroecosystems, such as biological control, highlighting both the high time-specific functional redundancy of the predator community, and temporal complementarity in prey use of the predator species.

## Materials and Methods

### Experimental set-up: study sites and collection of predators for MGCA

Our study was carried out in spring barley fields located in the region of Uppsala (59°51′N, 17°38′E), South central Sweden, in 2011. A total of 10 fields were selected as five pairs of organic (under organic management for more than 10 years) and conventional fields. The mean distance between organic and conventional fields was 1.6 km, ranging from 1.1 to 2.2 km, and the distance between the furthest pairs was 52.7 km. No effect of farming system on the abundances and prey detection rates of the same community of generalist predators analysed here were previously found, with the exception of a positive effect of farming system on the predation rate of carabids on spiders (families: Linyphiidae and Lycosidae)^42^. Because conventional farming systems in our study region are of low intensity^42,43^, we do not expect any differences in food web specialization between farming systems, and as such, only account for farming system in the random structure in our analyses.

The generalist predator community in this study was dominated in abundance by a few carabid and spider species: the carabids *Pterostichus melanarius* (Illiger)*, Poecilus cupreus* (Linnaeus)*, Harpalus rufipes* (Degeer)*, Bembidion lampros* (Herbst) and *Trechus secalis* (Paykull) represented 85% of the carabids and the spiders *Pardosa agrestis* (Westring, Lycosidae), *Oedothorax apicatus* (Blackwall, Linyphiidae) and *Agyneta rurestris* (Koch, Linyphiidae) represented more than 70% of the adult spiders caught by pitfall traps in the barley fields. For each of these eight species we caught at least eight individuals per field and all of them were present in all fields (except *T. secalis* who was missing from one field). All other predators present (Table S2) were caught in lower numbers, and they were missing from several fields. We therefore decided to focus our analyses on the above eight species. To examine their prey choices, we live-collected these arthropods at two distinct periods during the barley cropping season in 10 replicated fields using dry pitfall traps (11.5-cm diameter ×11-cm depth) containing clay balls (Weibulls, Åby, Sweden) and following recommended best practices^44^. Each week (weeks 22–23 and 25–26) within each period during the growing season, 12 to 35 traps (numbers adjusted depending on initial catches of predators to achieve a reasonable sample size of each target predator) were open for 24 h in each field. All predators were individually collected in 1.5-ml microtubes (Saarstedt, Nümbrecht, Germany), immediately frozen on dry ice, and stored at −80 °C until subsequent identification and DNA extraction. We screened the gut content of a total of 3,680 individual predators (Table S1) for various prey taxa using previously developed DNA-based molecular gut-content multiplex PCR assays^45^. These assays targeted the most abundant, and important extraguild prey groups for generalist predators in cereal crops of Northern and Central Europe^41^: aphids (>98% of aphids found in these fields were *Rhopalosiphum padi* with the rest being *Sitobion avenae*), dipterans, thrips, earthworms and springtails (Table 1). In addition, the predators were tested for 10 taxa of potential intraguild prey (including the screened predator taxa as well as lady beetles and lacewings common in these fields; Table 1). The screening was conducted as per the recommendations for molecular diagnostic work; see Method S1 and Table S3 for further details on sampling and bioassay specificity, material description and measures taken to prevent DNA contamination, see^42^. We pooled data from the two weeks within each period as the sample size from each individual week was considered too small to allow analysis at a higher temporal resolution. The number of individuals of each predator species caught per field and per time period varied from 0 to 117 (Table S1).

**Table 1:** Extraguild and intraguild prey targeted by our molecular assays (see^45^ for more details).

	Order	Family	Genera/Species	Common name
Extraguild prey	Hemiptera	Aphididae		Aphids
	Thysanoptera	Thripidae		Thrips
	Oligochaeta	Lumbricidae		Earthworms
	Arthropleona, Symphypleona			Springtails
	Diptera	Syrphidae, Anthomyiidae, Agromycidae, Calliphoridae, Chloropidae, Dolichopodidae, Drosophilidae, Empididae, Lonchopteridae, Muscidae, Rhagionidae, Tabanidae, Bibionidae, Cecidomyiidae, Sciaridae, Simulidae, Tipulidae, Trichoceridae		Dipterans
Intraguild prey	Coleoptera	Carabidae	*Pterostichus*	*Pterostichus*
	Coleoptera	Carabidae	*Poecilus*	*Poecilus*
	Coleoptera	Carabidae	*Harpalus*	*Harpalus*
	Coleoptera	Carabidae	*Bembidion*	*Bembidion*
	Aranea	Lycosidae		Wolf spiders
	Aranea	Linyphiidae		Sheet-web spiders
	Aranea	Tetragnathidae	*Pachygnatha*	*Pachygnatha*
	Coleoptera	Coccinellidae	*Coccinella septempunctata*	Seven-spot ladybird
	Neuroptera	Chrysopidae		Lacewings

We acknowledge that the trophic data generated is heterogeneous in terms of taxonomic resolution of the prey, with some being identified to order level (e.g., dipterans) and others being identified to family level (e.g., aphids, sheet-web spiders) (see above for a discussion of the potential implications of this). Trophic links detected by MGCA were used to build generalist predator-prey food webs for each field in the Early and Late period, using the detection frequencies (i.e., the percentage of predator individuals in which the prey was detected) as a measure of the strength of the link.

### Data treatment and analysis

#### Food web metrics

For each field, in the Early and Late period within the cropping season, we described food web specialization using quantitative, frequency-based, specialization metrics at both network- and species-level: web specialization, ${H^{\prime} }_{2}$ and predator specialization *d*’, respectively. A value for *d*’ was calculated for every predator species^20^. These indices are analogous to β-diversity of the distribution of feeding interactions at the network level (${H^{\prime} }_{2}$) and at the species levels (*d*’)^32^. Their values range from 0 for extreme generalization to 1 for extreme specialization. Both specialization metrics are independent of species frequency and sampling intensity, and are thus particularly well adapted for comparisons within (*d*’) and across food webs (${H^{\prime} }_{2}$)^20,32^. Specialization metrics were calculated in R^46^ using the functions ‘networklevel’ and ‘dfun’ in the statistical package *bipartite*^47^.

#### Food web randomization

We compared each observed feeding interaction matrix (20 matrices, based on the prey DNA detection frequencies) to null model expectations. This null model approach makes the minimalistic assumption that each observed predation event in a field and season represents prey that have been available to all predators present in that field and that these predators sample these ‘available’ prey at random without any preference. More specifically, 1000 random null model replicates were generated by our own algorithm in R (Method S2) that randomly redistributes the observed feeding interactions of a prey among the allowable predators (specifically avoiding ‘forbidden’ links inherent to the molecular methods used, i.e., the impossibility of detecting cannibalism, as well as intraguild predation within the same genus for carabids and the same family for spiders). The redistribution of observed feeding interactions among predators was made according to a multinomial distribution with probabilities proportional to the number of predator individuals analysed for the different predator species while keeping both the row sums (total frequency of detection of prey taxa) and column sums (frequency of detection per predator species) constant (i.e., equal to the ones in the observed feeding interaction matrix). As a result, in every null model replicate, each predator species and each prey taxon was assigned the same number of total feeding interactions (prey items and predation events respectively) as observed in the empirical data, but with a variable distribution of detection frequencies (counts) among its prey and predator species.

We defined link temperature *T*_*i,j,k*_ between prey *i* and predator *j* at site *k* as:

1 $${T}_{i,j,k}=\frac{{f}_{{1}_{i,j,k}}-{f}_{{2}_{i,j,k}}}{1000}$$

With *f*_*1 i,j,k*_ the number of randomly generated null model webs, for web *k*, with a feeding interaction frequency (count) between prey *i* and predator *j* smaller than the observed interaction frequency (count), and *f*_*2 i,j,k*_ the number of null model webs with a feeding interaction frequency (count) greater than the observed. This constrains *T*_*i,j,k*_ between −1 and 1, where *T*_*i,j,k*_ > 0 means that the observed feeding interaction occurs more often than the majority of null model frequencies, and *T*_*i,j,k*_ < 0 means that the observed feeding interaction occurs less often than the majority of null model frequencies. Warm and cool links between prey *i* and predator *j* are thus defined by *T*_*i,j*_ > 0 and *T*_*i,j*_ < 0, respectively.

We defined the significance level of each observed link temperature by using a bootstrapping approach where we used the existing null model webs to generate a distribution of random link temperatures *T*_*R,i,j,k*_ between prey *i* and predator *j* at site *k*. More specifically, at each site *k*, we sampled one web from the distribution of 1000 null model webs, and calculated the link temperature for each link. We repeated this procedure 1000 times to get a distribution of *T*_*R,i,j,k*_. We calculated *F*_*i,j,k*_, the frequency (proportion) of random link temperatures (proportion) *T*_*R,i,j,k*_ smaller or greater than the observed link temperature *T*_*i,j,k*_. For warm and cold links, proportions >0.95 and <−0.95 were chosen to signify a significant deviation at a site. Furthermore, as a measure of the overall deviance from null model expectations for each trophic link between prey *i* and predator *j* across all sites, we used binomial tests that revealed significant deviation(s) present in the data by providing the probability of obtaining the observed number of sites (assuming a random distribution of feeding interactions and thus a probability of 0.95 of conforming to null model expectations at each site).

### Statistical analyses

To analyse the variation within the cropping season in (i) the observed food web metrics, and (ii) the deviation in these metrics from null model expectations, we used linear mixed effect models testing each metric and their deviation against within-season changes only (‘Early’ *vs* ‘Late’) (for web specialization); or within-season changes and the effects of predator species and their two-way interactions (for species-level specialization). The response variable was:

(i) ${X}_{obs{}_{i}}$, the observed metrics at site *i*,
(ii) $\Delta i\,=\,{X}_{obs{}_{i}}\,-\overline{{X}_{exp{}_{i}}}$ with $\overline{{X}_{exp{}_{i}}}$ the expected metric for the food web at site *i*, assuming random distribution of observed feeding interactions (calculated as the mean metric of 1000 null model replicates).

The random effects accounted for temporal (“field”) and spatial (“field pair”) autocorrelations inherent to the experimental design and additionally accounts for paired field management (organic vs conventional). To test the effects of time periods within the cropping season, predator species, and prey taxa on the deviation from random (i.e., zero) of the temperature of links, we used the absolute value of link temperatures as the response variable, and time periods, predator species, prey taxa, and all interactions among these variables in a linear mixed effect model with the same random structure as described above for the food web metrics. The absolute values of the temperature of links were arcsine-square root transformed to meet the assumption of normality. We simplified all models using the Akaike Information Criterion (Δ < 2). The ‘lmer’ function of the lme4 package^48^ was used for these analyses. The ANOVA results are presented in Tables S4–S6. To analyse if the observed metrics were different from null model expectations at each site, we calculated the proportion of 1000 null model replicates with values lower than the observed value. Proportions of <0.025 or >0.975 were chosen to signify a significant deviation at a site. Furthermore, as a measure of the overall deviance from null model expectations across all sites, we used binomial tests that, for each metric, provided the probability of obtaining the observed number of sites that showed a significant deviation (assuming a random distribution of feeding interactions and thus a probability of 0.95 of conforming to null model expectations at each site).

### Data availability

The molecular gut content analyses of predators analysed in this manuscript will be deposited in figshare.

## Electronic supplementary material

Supplementary Information
