# Supplementary material for: High Redundancy as well as Complementary Prey Choice Characterize Generalist Predator Food Webs in Agroecosystems
Source: Sci Rep. 2018 May 23;8:8054. doi: 10.1038/s41598-018-26191-0 (PMC5966386; doi:10.1038/s41598-018-26191-0)
Supplement: Supplementary file 1 — Supplementary Information [file 41598_2018_26191_MOESM1_ESM.pdf]

High Redundancy as well as Complementary Prey Choice Characterize Generalist Predator Food Webs in Agroecosystems

Eve Roubinet [a], Tomas Jonsson [a, b], Gerard Malsher [a], Karin Staudacher [c], Michael Traugott [c], Barbara Ekbom [a], Mattias Jonsson [a*]

[a] Department of Ecology, Swedish University of Agricultural Sciences, Uppsala, Sweden

[b] Ecological Modelling group, School of Biosciences, Skövde University, Skövde, Sweden

[c] Mountain Agriculture Research Unit, Institute of Ecology, University of Innsbruck, Innsbruck, Austria

[*] Corresponding author: mattias.jonsson@slu.se

Supplementary information

Table S1: Number of predator screened per field and per period during the cropping season.

Table S2: Predator taxa caught in the pitfall traps.

Table S3a-c: Final concentration of primers when used in the diagnostic multiplex PCRs.

Fig. S1: Proportion of predator individuals screened that were positive for a) extraguild prey and b) intraguild prey in eight generalist predator species.

Table S4: ANOVA table showing the effects of period on the food-web metrics.

Table S5: For network-level specialization, p-value for each site as the proportion of 1000 null model replicates with values lower than the observed value, and p-value from binomial tests as a measure of the overall deviance from null model expectations across all sites.

Table S6: ANOVA table showing the effect of period on the deviation of the food-web metrics to the mean expected from 1000 null models.

Table S7: For species-level specialization, p-value for each site as the proportion of 1000 null model replicates with values lower than the observed value. On the top row, p-value from binomial tests as a measure of the overall deviance from null model expectations across all sites.

Table S8: ANOVA table showing the effect of period on the absolute value of the temperature of links.

Fig. S2: Extraguild prey density in the Early and Late period within the cropping season.

Method S1: Protocol explaining the DNA-based MGCA of field-collected predators.

Method S2: Algorithm in R to generate null model that randomly redistributes the observed feeding interactions of a prey among the allowable predators

Table S1: Number of predators (carabids: *Pterostichus melanarius, Poecilus cupreus, Harpalus rufipes, Bembidion lampros, Trechus secalis* and spiders: *Agyneta rurestris* [Linyphiidae], *Oedothorax apicatus* [Linyphiidae] and *Pardosa agrestis* [Lycosidae]) screened per field and per period (Early, Late) within the cropping season. Fields 1-5 (in grey) and 6-10 were managed conventionally and organically, respectively.

	Field	***P. mel***	***P. cup***	***H. ruf***	***B. lam***	***T. sec***	***A. rur***	***O. api***	***P. agr***
Early period	1	8	16	57	1	16	21	5	3
	2	0	45	36	21	42	3	52	36
	3	12	6	48	28	3	4	4	1
	4	10	4	2	5	0	31	18	3
	5	50	3	48	36	15	2	4	59
	6	50	48	32	11	33	31	13	56
	7	15	15	23	2	0	0	51	58
	8	32	42	23	5	1	12	14	7
	9	1	50	37	41	5	10	30	50
	10	23	11	14	16	4	11	12	52
Late period	1	50	28	31	37	8	22	8	2
	2	4	74	37	39	6	3	31	8
	3	21	1	67	36	3	8	5	0
	4	42	3	37	26	0	0	51	2
	5	117	0	75	56	7	0	5	17
	6	49	41	50	20	11	1	5	16
	7	28	14	24	27	1	2	20	51
	8	50	50	50	49	0	5	20	6
	9	17	29	49	41	5	1	32	8
	10	50	14	48	50	4	8	18	50
**Total**	**-**	**629**	**494**	**788**	**547**	**164**	**175**	**398**	**485**

Table S2: Predator taxa caught in the pitfall traps. In addition to these several specimens of Staphylinidae were caught. However, they were not determined to species.

**Araneae**	**Coleoptera**
Pardosa agrestis	*Harpalus rufipes*
Oedothorax apicatus	*Pterostichus melanarius*
Meioneta rurestris	*Bembidion lampros*
Trochosa sp.	*Poecilus cupreus*
Pardosa palustris	*Trechus secalis*
Linyphiidae sp.	*Bembidion quadrimaculatum*
Erigone atra	*Trechus quadristriatus*
Pardosa amentata	*Poecilus versicolor*
Pardosa prativaga	*Harpalus affinis*
Trochosa ruricola	*Pterostichus niger*
Porrhomma microphthalmum	*Bembidion guttula*
Pardosa sp.	*Harpalus seladon*
Erigone dentipalpis	*Harpalus tardus*
Trochosa spinipalpis	*Trechus discus*
Oedothorax retusus	*Harpalus distinguendus*
Pardosa fulvipes	*Coccinella septempunctata*
Pardosa pullata	
Micrargus subaequalis	
Porrhomma pygmaeum	
Walckenaeria nudipalpis	

Table S3a: Targets, primer names, amplicon length and final concentration (Conc.; * indicates the reduced primer concentration when screening the respective consumer, i.e., beetle or spider) of each group-specific primer (forward and reverse) when used in the "MPI" PCRs. This table was originally published in[42].

Targets	Primer names (forward-reverse)	Amplicon length (bp)	Conc. (µM)
beetles/thrips	S405-A406	~208	0.2 (0.04*)
Spiders	S407-A408	~258	0.2 (0.04*)
Aphids	S423-A424	148	0.4
Earthworms	S408-A413	85	0.2
Springtails	S411-A415	289	0.2
Dipterans	S414-A416	341	0.4
Lacewings	S417-A420	390	0.2

Table S3b: Targets, primer names, amplicon length and final concentration (Conc.) of each primer (forward and reverse; if concentrations are different, both are listed) when used in the "MPII spiders" PCRs.

Targets	Primer names (forward-reverse)	Amplicon length (bp)	Conc. (µM)
Lycosidae	S486-A488	181	0.4 and 0.6
Linyphiidae	S487-A490	153	0.4
Pachygnatha	S488-A493	249	0.6 and 0.8
Springtails	S411-A415	289	0.1

Table S3c: Targets, primer names, amplicon length and final concentration (Conc.) of each primer (forward and reverse; if concentrations are different, both are listed) when used in the "MPII beetles/thrips" PCRs.

Targets	Primer names (forward-reverse)	Amplicon length (bp)	Conc. (µM)
Poecilus	S475-A486	112	0.15
Pterostichus	S467/S467.1-A467.1	166	0.4 and 0.2
Bembidion	S468-A470	~152	0.15 and 0.1
Harpalus	S473-A475	349	0.6 and 0.55
Thrips	S477-A481	~272	0.2 and 0.4
Coccinella septempunctata	S480-A485	238	0.15 and 0.1

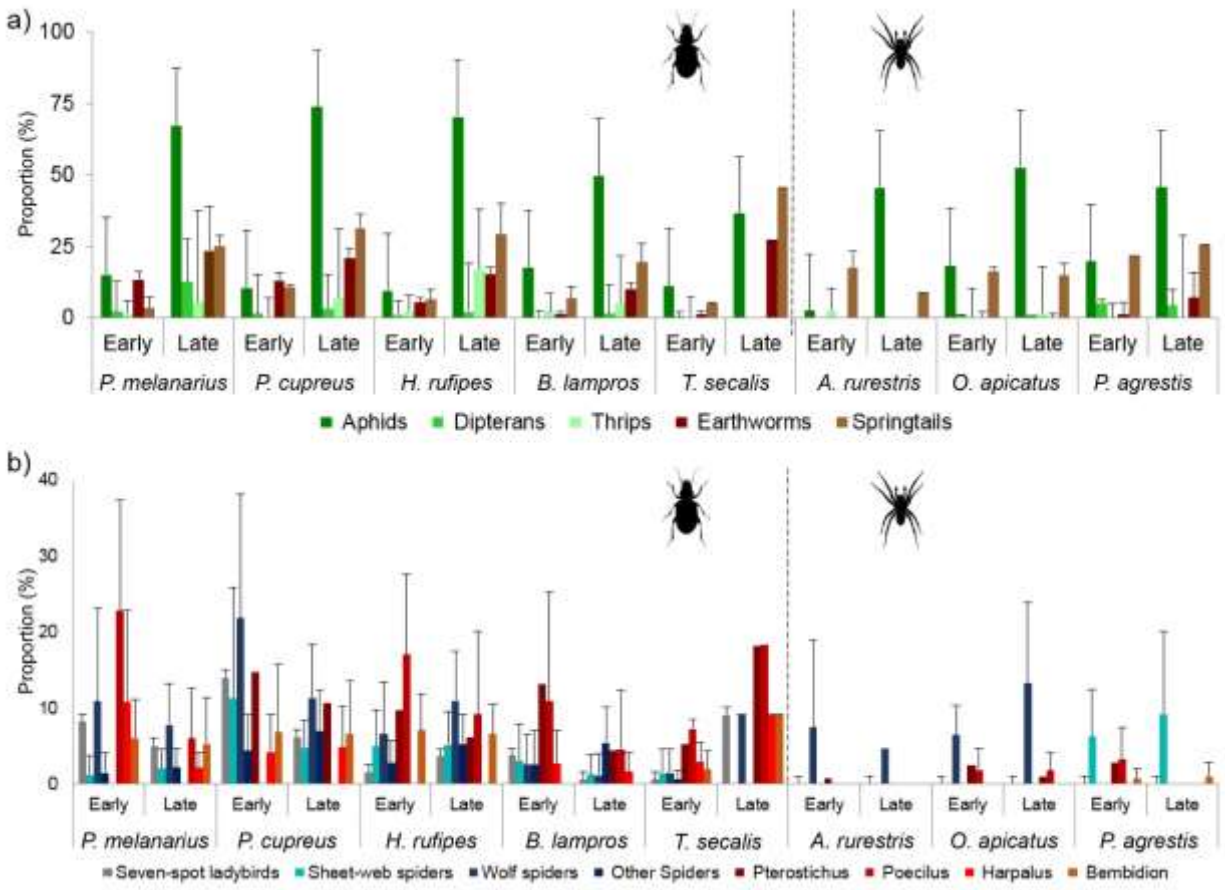

Fig. S1: Proportion of predator individuals screened that were positive for a) extraguild prey and b) intraguild prey in eight generalist predator species (carabids: *Pterostichus melanarius*, *Poecilus cupreus*, *Harpalus rufipes*, *Bembidion lampros*, *Trechus secalis* and spiders: *Agyneta rurestris* [Linyphiidae], *Oedothorax apicatus* [Linyphiidae] and *Pardosa agrestis* [Lycosidae] in the Early and Late period during the cropping season (mean ± SD detection frequencies in individual fields). Predators were included in the calculation if the number of screened individuals >9/field/period. Prof Klaus Birkhofer is acknowledged for drawing the invertebrates.

Table S4: ANOVA table showing the effects of period within the cropping season on the food-web metrics. Bold letters highlight significant factors (α=0.05). Italicized interactions were removed during model selection based on AIC.

	Factor	Mean Sq	NumDF	DenDF	F.value	Pr(>F)
H_2	**Period**	**0.14**	**1**	**9**	**53.43**	**<0.001**
d’	Period	0.69	1	117.32	41.2	<0.001
	Predator	0.03	7	117.43	1.54	0.16
	**Period:Predator**	**0.05**	**7**	**117.32**	**3.27**	**0.003**

Table S5: For network-level specialization, p-value for each site (numbered 1 to 10) as the proportion of 1000 null model replicates with values lower than the observed value (Prop) in the Early and Late period within the cropping season (light and dark grey cases respectively). P-values lower than 0.025 or larger than 0.975 are highlighted in yellow. On the last column, p-value from binomial tests (in bold if lower than 0.05) as a measure of the overall deviance from null model expectations across all sites (see methods).

	Field										**Binom**
	1	2	3	4	5	6	7	8	9	10	**. Test**
Prop. Early	0.882	0.996	0.946	0.046	0.987	0.998	0.853	0.846	0.616	0.069	**0.012**
Prop. Late	1	1	0.979	0.572	0.918	1	0.641	0.994	0.985	1	**0.001**

Table S6: ANOVA table showing the effect of period within the cropping season on the deviation of the food-web metrics to the mean expected from 1000 null models. Bold letters highlight significant factors (α=0.05). Italicized interactions were removed during model selection based on AIC.

	Factor	Mean Sq	NumDF	DenDF	F.value	Pr(>F)
Δ H_2'	Period	0.01	1	9	3.53	0.092
Δ d'	Period	0.08	1	124.11	3.38	0.068
	**Predator**	**0.14**	**7**	**124.41**	**6.11**	**<0.001**
	Period:Predator	*0.04*	*7*	*117.26*	*1.75*	*0.104*

Table S7: For species-level predator specialization, p-value for each site (numbered 1 to 10) as the proportion of 1000 null model replicates with values lower than the observed value in the Early and Late period (light and dark grey cases respectively). P-values lower than 0.025 or larger than 0.975 are highlighted in yellow. On the top row, p-value from binomial tests (in bold if lower than 0.05) as a measure of the overall deviance from null model expectations across all sites (see methods). The eight generalist predators are: carabids: *Pterostichus melanarius, Poecilus cupreus, Harpalus rufipes, Bembidion lampros, Trechus secalis* and spiders: *Agyneta rurestris* (Linyphiidae), *Oedothorax apicatus* (Linyphiidae) and *Pardosa agrestis* (Lycosidae). NA denotes field where no individuals of the particular predator species have been screened.

		P. mel	***P. cup***	***H. ruf***	***B. lam***	***T. sec***	***A. rur***	***O. api***	***P. agr***
**Binom. test**		P=0.063	P=1.000	P=0.074	P=1.000	**P<0.001**	P=0.063	P=0.063	**P<0.001**
Field	1	0.902	0.740	0.449	NA	0.992	0.017	0.837	0.696
	2	NA	0.947	0.679	0.626	1.000	0.736	0.325	0.982
	3	0.765	0.347	0.995	0.633	NA	0.022	0.669	0.591
	4	0.011	0.169	0.342	0.556	NA	0.505	0.293	0.000
	5	0.717	0.792	0.992	0.733	1.000	0.568	NA	0.633
	6	0.786	0.948	0.282	0.953	1.000	0.648	0.858	0.024
	7	0.992	0.107	0.386	0.875	NA	NA	0.973	0.008
	8	0.895	0.859	0.559	NA	NA	0.487	0.006	0.470
	9	0.628	0.091	0.954	0.121	1.000	0.272	0.800	0.414
	10	0.244	0.274	0.169	0.363	0.976	0.399	0.290	0.025
**Binomials test**		**P<0.001**	**P<0.001**	**P<0.001**	P=0.310	**P<0.001**	P=0.257	**P=1.000**	P=0.289
Field	1	1.000	0.997	0.996	0.966	1.000	0.997	0.589	0.132
	2	0.997	0.999	0.998	0.551	1.000	0.824	0.944	0.668
	3	0.967	0.569	0.941	0.770	0.958	0.094	NA	NA
	4	0.259	0.688	0.144	0.319	NA	NA	0.727	0.434
	5	0.508	NA	0.106	0.971	0.653	NA	0.631	0.993
	6	1.000	0.999	0.999	0.936	1.000	NA	0.516	0.444
	7	0.492	0.964	0.842	0.270	0.372	0.963	0.054	0.255
	8	0.942	0.909	0.804	0.998	NA	0.703	0.954	0.642
	9	0.975	0.994	0.327	0.327	1.000	0.709	0.032	0.813
	10	1.000	1.000	1.000	0.866	NA	0.271	0.205	0.762

Table S8: ANOVA table showing the effect of period within the cropping season on the absolute value (i.e., the deviation from zero) of the temperature of links (arcsine square-root transformed). Bold letters highlight significant factors (α=0.05). Italicized interactions were removed during model selection based on AIC.

	Sum Sq	Mean Sq	NumDF	DenDF	F.value	Pr(>F)
Period	0.06	0.06	1	1390.2	0.30	0.586
**Predator**	**35.10**	**5.01**	**7**	**1390.2**	**24.46**	**<0.001**
**Prey**	**16.35**	**1.36**	**12**	**1389.2**	**6.65**	**<0.001**
**Predator:Prey**	**42.21**	**0.58**	**73**	**1388.2**	**2.82**	**<0.001**
Period:Prey	*2.50*	*0.21*	*12*	*1297.6*	*1.02*	*0.427*
Period: Predator	*2.69*	*0.38*	*7*	*1299.0*	*1.88*	*0.069*
Period: Predator: Prey	*14.53*	*0.20*	*72*	*1297.1*	*0.99*	*0.505*

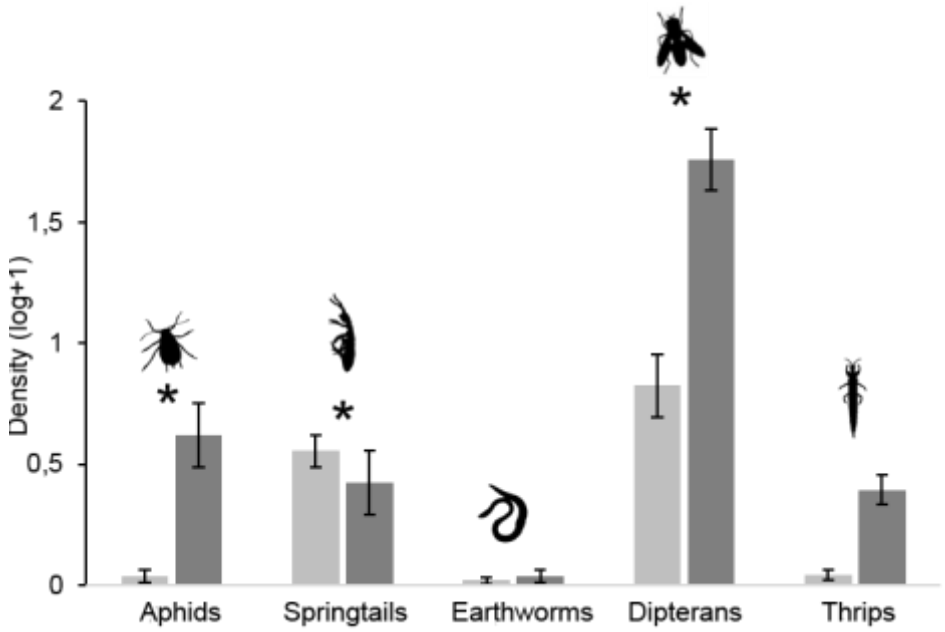

Fig. S2: Extraguild prey density (mean per sample unit (log x+1 transformed)/per field ± SEM) in the Early (light grey) and Late period (dark grey) of the cropping season. * denotes significant within seasonal variations (α=0.05) using permutational analyses of variance for a hierarchical mixed effects model with fixed factors farming system and period within the cropping season for extraguild prey abundances (log x+1 transformed) (Roubinet *et al.* 2017). Prof Klaus Birkhofer is acknowledged for drawing the invertebrates.

Reference:

Roubinet, E., K. Birkhofer, G. Malsher, K. Staudacher, B. Ekbom, M. Traugott, and M. Jonsson. 2017. Diet of generalist predators reflects effects of cropping period and farming system on extra- and intraguild prey. Ecological Applications 27:1167–1177.

Method S1: After morphological species identification each predator sample was subjected to whole-body DNA extraction and screened with diagnostic PCR assays largely following the protocols described in[45] with some modifications (see below and for a detailed description[42]). A first group-specific multiplex PCR assay ("MPI") allowed the examination of a predator's prey choice on a more general level (targeting extraguild: aphids, dipterans, springtails, and earthworms; and intraguild prey groups: lacewing, spiders, beetles/thrips) followed by two additional assays enabling the assessment of IGP at a higher taxonomic resolution ("MPII spiders": Lycosidae, Linyphiidae, *Pachygnatha*, and "MPII beetles/thrips": thrips, *Pterostichus*, *Poecilus*, *Harpalus*, *Bembidion*, *Coccinella septempunctata*).

The "MPI" assay was performed in a total volume of 10 µl containing 1.5 µl of DNA extract, 1× QIAGEN Multiplex PCR Master Mix (Qiagen), each primer at its corresponding concentration (Table S2a), 0.5× Q-solution (Qiagen), 5 µg BSA, 30 mM TMAC (Sigma-Aldrich), and PCR-grade water to adjust the volume. Amplifications were carried out under the following thermocycling conditions: 15 min at 95°C, 35 cycles of 30 s at 94°C, 90 s at 60°C and 90 s at 72°C, and 10 min at 72°C.

The "MPII spiders" assay was performed in 10 µl PCRs containing 1.5 µl of DNA extract, 1× Type-it Multiplex PCR Master Mix (Qiagen), each primer at its corresponding concentration (Table S2b), and 5 µg BSA. The thermocycling protocol included an initial activation step of 5 min at 95°C, followed by 35 cycles of 30 s at 95°C, 90 s at 63.5°C and 30 s at 72°C, and 10 min at 68°C. Not all beetles testing positive for spider-prey in "MPI" could be assigned to a specific spider taxon (i.e.,

lower taxonomic level) in "MPII spiders". These predator DNA samples were subsequently subjected to DNA barcoding to resolve feeding interactions that occurred with other spider taxa than the targeted Lycosidae, Linyphiidae, and *Pachygnatha*. All barcoded spider-prey samples were assigned to family level to make results comparable to the ones of the “MPII spiders” assay.

The PCR protocol of the “MPII beetles/thrips” assay differed only slightly from the “MPII spiders”: 1.5 µl of DNA extract and 30 mM TMAC (Sigma-Aldrich) were used in the total volume of 10 µl (plus PCR-grade water to adjust the volume); for primer concentrations see Table S2c. Thermocycling conditions were as described above, but with an annealing temperature of 63.5°C. A detailed description of primer sequences and assay specificity can be found in[45]. All PCR products were separated and visualized using the QIAxcel electrophoresis system (Qiagen) following the protocol described in[45].

Method S2: Script to generate random null-model versions of an observed matrix with integer frequencies. The random matrices are filled row by row, assuming that the observed frequencies are multinomially redistributed among the allowed entries, making sure that the row sums and column sumsnstay the same as in the observed matrix. Forbidden entries (e.g. impossible links between predator and prey) should be coded by -1 in the observed matrix.

The necessary inputs are:

- n_repl: number of null-model replicates to generate
- InMat: observed matrix with integer frequencies
- attempt_max: maximum number of iterations to try for obtaining the desired number of replicates. Obviously, attempt_max > n_repl (e.g. set attempt_max to 10 or 100 times n_repl)

The output is a list of random matrices (Rand_mats).

The script has been written by Tomas Jonsson (25 January 2016).

_____

```
randmats_multinomial2 = function(n_repl,InMat,pred_num,attempt_max) {
#----------------------------------------
#INITIALIZATION:
#Check that desired number of replicates does not exceed number of iteration
attempts:
if (attempt_max < n_repl) {
        stop('Number of iteration attempts can not be smaller than the desired number
of replicates!')
}

```

```
199 #Create working matrix with coding for forbidden links removed:
200 InMat2 = InMat;
201 InMat2[InMat==-1] = 0; #remove -1 from observed matrix and replace with zeros:
202
203 #Obtain web statistics:
204 row_sums = rowSums(InMat2); #row sums
205 col_sums = colSums(InMat2); #column sums
206 n_obs = sum(InMat2); #total number of observations
207 n_rows = length(row_sums); #number of rows
208 n_cols = length(col_sums); #number of columns
209
210 #Initialize list for storing random matrices:
211 Rand_mats = vector('list',n_repl);
212
213 #By default, the random number generator (RNG) is seeded based on the current time
214 so that rand produces a different sequence of numbers after each time rng is called. If
215 the same random number sequence is wanted, use set.seed to set RNG to a known
216 state, e.g.: set.seed(12)
217
218 repl = 0; #set counter for number of successful replicates to zero
219 attempts = 0; #set counter for number of iteration attempts to zero
220
221 # Obtain loop order for rows (first all-zero-rows, then rows with forbidden links
222 sorted in descending order of number of forbidden links, followed by rows with
223 nonzero elements and no forbidden links):
```

```
all_zeros_ri = which(row_sums==0) #rows with all zero elements (no non-zero
entries)
A_temp = matrix(0,nrow=n_rows,ncol=n_cols); A_temp[InMat==-1]=1 #temporary
matrix with position of forbidden links coded by one...
row_sums_A_temp = rowSums(A_temp); #Number of forbidden links per row
col_sums_A_temp = colSums(A_temp); #Number of forbidden links per column
sort_res = sort(row_sums_A_temp,decreasing = TRUE,index.return = 1) #obtain sort
order for rows in descending order after number of forbidden links
r = sort_res$x; fl_sort_i = sort_res$ix
nonzero_ri = setdiff(fl_sort_i,all_zeros_ri);
row_order= c(all_zeros_ri, nonzero_ri); #loop through rows in this order

#----------------------------------------
#MAIN ALGORITHM:
while (repl < n_repl) { #Outer loop for generating a set of random matrices
        next_attempt_flag = 0; #Flag used to jump to next iteration attempt
        attempts = (attempts + 1); #update number of iteration attempts

        #Initialize random matrix and necessary parameters:
        rnd_mat = matrix(0,nrow=n_rows,ncol=n_cols);
        rem_colfreq = col_sums; #Remaining column frequencies (i.e. number of
observations remaining to distribute in columns)
        rem_rowfreq = row_sums #Remaining row frequencies (i.e. number of
observations remaining to distribute on rows)
```

```
        rem_obs = n_obs; #Total number of observations remaining to distribute in
matrix

        #Main loop for allocating random numbers to matrix:
        for (r_ind in 1:(n_rows-1)) { #loop through all but "last" row:
                if (row_sums[row_order[r_ind]] !=0) { #ignore rows with no non-zeros
entries!
                        #Obtain loop order for elements (columns) on row in question
(i.e. row_order[r_ind])):
                        all_zeros_ci = which(col_sums==0) #columns with no non-
zeros entries
                        fl_ind1 = which(InMat[row_order[r_ind],] == -1); #elements
with forbidden links on row in question
                        fl_ind2 = which(InMat[row_order[r_ind+1],] == -1); #elements
with forbidden links on next row
                        c_ind_temp1 = sort(union(all_zeros_ci,fl_ind1)); #union of
elements with column sum equal to zero and elements with forbidden links on row in
question...
                        c_ind_temp2 = sort(union(c_ind_temp1,fl_ind2)); #union of
elements with column sum equal to zero and elements with forbidden links on this and
next row...
                        c_ind_temp3 = setdiff((1:n_cols),c_ind_temp2); #Extract
indices in 1:n_cols that are not in c_ind_temp2
                        col_order = c(c_ind_temp2, c_ind_temp3); #loop through
elements on row l in this order
```

```
                    #Generate multinomially distributed random numbers:
                    multinom_nn = length(c_ind_temp3) #number of allowed
entries on row l (i.e. number of potentially non-zero elements)
                    multinom_size = row_sums[row_order[r_ind]] #total number
of frequencies to distribute on row l
                    #multinom_prob = rep(1,multinom_nn) #relative probabilities
for elements
                    multinom_prob = pred_num/sum(pred_num)
                    multinom_freq = rmultinom(1:multinom_nn , multinom_size,
multinom_prob) #multinomially distributed random numbers!
                    for (c_ind in 1:(n_cols-1)) { #loop through all but "last"
element (column) of row l
                                #Check that element (i) is not in a column with initial
or (ii) does not have remaining column sum equal to zero and (iii) that element does
not forbidd non-zero entry:
                                if (col_sums[col_order[c_ind]] != 0) { #ignore elements
with column sum equal to zero!
                                        if (rem_colfreq[col_order[c_ind]] != 0) {#ignore
elements with remaining column sum equal to zero
                                                if
(InMat[row_order[r_ind],col_order[c_ind]] != -1) { #Ignore elements that forbidd
non-zero entry
                                                        #Check whether all forthcoming
elements in this column are forbidden, if so put all remaining observations for this
column here!
```

```
                                        if
(all(InMat[row_order[(r_ind+1):n_rows],col_order[c_ind]] == -1)) {
        rnd_mat[row_order[r_ind],col_order[c_ind]] = rem_colfreq[col_order[c_ind]];
        rem_rowfreq[row_order[r_ind]] = rem_rowfreq[row_order[r_ind]] -
rem_colfreq[col_order[c_ind]] #Update remaining number of obs to distribute on row
l
                                            rem_obs = rem_obs -
rem_colfreq[col_order[c_ind]]
        rem_colfreq[col_order[c_ind]] = 0
                                        } else {
                                            if
(rem_rowfreq[row_order[r_ind]] == 0) {#row is full
                                                break #terminate
(break out of) c_ind loop!
                                            }
                                            #Allocate randomly
picked multinomially distributed number within interval set by constraints to (i) not
exceed remaining column or rowsum for element in question, and (ii) be able to
reach rowsum with remaing columnsums:
                                            rnd_lmn =
multinom_freq[col_order[c_ind]] #
                                            rnd_max =
min(rem_colfreq[col_order[c_ind]],rem_rowfreq[row_order[r_ind]]); #maximum
value for random number that does not exceed remaining column or rowsum
frequencies
```

```
                                                    rnd_min =
rem_rowfreq[row_order[r_ind]] - sum(rem_colfreq[col_order[(c_ind+1):n_cols]]);
#minimum value needed for random number in order for remaining rowsum to be
reached with remaining columnsums
                                                    if (rnd_min < 0) {
                                                            rnd_min = 0;
                                                    }
                                                    if (rnd_min > rnd_max)
{#
                                                            warning('Negative
interval attempted for random number generator!')
                                                            next_attempt_flag
= 1;
                                                            break #terminate
(break out of column loop: for c_ind = 1:(n_cols-1))
                                                    } else {#
                                                            if (rnd_min ==
rnd_max) {
                                                                    rnd_lmn =
rnd_min; #frequency can only take on this value!
                                                            } else {#rnd_min
< rnd_max
                                                                    while
(rnd_lmn>rnd_max) {#
                rnd_lmn = rnd_lmn-1
```

```
348                                                                 }
349                                                                 while
350 (rnd_lmn<rnd_min) {#
351             rnd_lmn = rnd_lmn+1
352                                                                 }
353                                                             }
354                                                         }
355
356                                                         #Check whether
357 suggested random element fulfils remaining criteria, if not make corrections...
358                                                         z = 1;
359                                                         while (z == 1) {#
360                                                             w1 = rem_obs -
361 rnd_lmn; #total number of observations remaining to distribute in matrix if rnd_lmn
362 is accepted
363                                                             w2 = rem_colfreq;
364             w2[col_order[c_ind]] = w2[col_order[c_ind]] - rnd_lmn; #remaining column
365 frequencies if rnd_lmn is accepted
366                                                             w3 = sum(w2);
367 #total remaining observations that can be distributed in "available" elements based
368 on remaining column frequencies
369                                                             if (rnd_lmn >
370 rnd_max) {
371             warning('Algorithm failure, next attempt...')
372             next_attempt_flag = 1;
```

```
373                                                     break
374     #terminate (break out of) while loop
375                                             }
376                                             if (rnd_lmn <
377     rnd_min) {
378             warning('Algorithm failure, next attempt...')
379             next_attempt_flag = 1;
380                                                     break
381     #terminate (break out of) while loop
382                                             }
383
384                                             #Is suggested
385     random number too large or too small?
386                                             if (w1 < w3) {
387                                                     rnd_lmn =
388     rnd_lmn - 1;
389                                             } else {
390                                                     if (w1 >
391     w3) {
392             rnd_lmn = rnd_lmn + 1;
393                                                     } else
394     {#w1 == w3
395             break
396                                                     }
397                                             }
```

```
                                                    } #end of while loop
(z==1)
                                                    if (next_attempt_flag ==
0) {
        rnd_mat[row_order[r_ind],col_order[c_ind]] = rnd_lmn; #element m of row l
        rem_rowfreq[row_order[r_ind]] = rem_rowfreq[row_order[r_ind]] - rnd_lmn;
#Update remaining number of obs to distribute on row l
        rem_colfreq[col_order[c_ind]] = rem_colfreq[col_order[c_ind]] - rnd_lmn;
#Update remaining number of obs to distribute in column m
                                                        rem_obs =
rem_obs - rnd_lmn; #Update total number of remaining observations to distribute
                                                    }
                                                }
                                            }
                                        }
                                    }
                                } #end of column loop (for c_ind in 1:(n_cols-1))
                                if (next_attempt_flag == 1) {
                                    break
                                } else {
                                    if (rem_rowfreq[row_order[r_ind]] != 0) {
                                        rnd_mat[row_order[r_ind],col_order[n_cols]] =
rem_rowfreq[row_order[r_ind]]; #last element (column) on row in question
```

```
421                                     rem_colfreq[col_order[n_cols]] =
422 rem_colfreq[col_order[n_cols]] - rem_rowfreq[row_order[r_ind]]; #Update
423 remaining number of obs to distribute in column in question
424                                     rem_obs = rem_obs -
425 rem_rowfreq[row_order[r_ind]]; #Update total number of remaining observations to
426 distribute
427                                     rem_rowfreq[row_order[r_ind]] = 0; #Update
428 remaining number of obs to distribute on row in question
429                                 }
430                             }
431                         }
432             } #end of row loop (for r_ind in 1:(n_rows-1))
433             if (next_attempt_flag == 0) {#
434                 #Compute entries of "last" row of rnd_mat:
435                 ind_lr = which(InMat[row_order[n_rows],] != -1); #loop through
436 elements on last row in this order
437                 #ind_lr = sample(ind_lr) #randomly permute order of elements
438                 for (c_ind in 1:(length(ind_lr)-1)) {
439                     if (InMat[row_order[n_rows],ind_lr[c_ind]] != -1) { #Check
440 that element does not forbidd non-zero entry
441                         rnd_mat[row_order[n_rows],ind_lr[c_ind]] =
442 rem_colfreq[ind_lr[c_ind]];
443                         rem_rowfreq[row_order[n_rows]] =
444 rem_rowfreq[row_order[n_rows]] - rem_colfreq[ind_lr[c_ind]];
```

```
                                rem_obs = rem_obs - rem_colfreq[ind_lr[c_ind]];
#Update total number of remaining observations to distribute
                                rem_colfreq[ind_lr[c_ind]] = 0;
                        }
                }
                #last element of last row:
                rnd_mat[row_order[n_rows],ind_lr[length(ind_lr)]] =
rem_rowfreq[row_order[n_rows]];
                #Make final check that row and column sums match observed
matrix:
                row_sums_rnd = rowSums(abs(rnd_mat)); #row sums of absolute
element values in random matrix
                col_sums_rnd = colSums(abs(rnd_mat)); #column sums of absolute
element values in random matrix
                if (all(all(row_sums_rnd == row_sums) && all(col_sums_rnd ==
col_sums))) {
                        repl = (repl + 1);
                        Rand_mats[[repl]] = rnd_mat;
                }
        }
        if (attempts > attempt_max) {
                message('Maximum number of attempts (',attempt_max,') exceeded!')
                break
        }

```

```
}
message('Number of attempts = ',attempts)

#Collect Output (random matrices...):
return(list(Rand_mats))
}
```
